# Immune Profiling of Vulvar Squamous Cell Cancer Discovers a Macrophage-rich Subtype Associated with Poor Prognosis

**DOI:** 10.1158/2767-9764.CRC-22-0366

**Published:** 2024-03-21

**Authors:** Mateja Condic, Andrea Rohr, Soheila Riemann, Christian Staerk, Tiyasha H. Ayub, Anna Doeser, Thomas Zillinger, Sabine Merkelbach-Bruse, Reinhard Buettner, Winfried Barchet, Christian Rudlowski, Alexander Mustea, Kirsten Kübler

**Affiliations:** 1 Department of Gynecology and Gynecological Oncology, University Hospital Bonn, Bonn, Germany.; 2 Institute of Clinical Chemistry and Clinical Pharmacology, University Hospital Bonn, Bonn, Germany.; 3 Department of Medical Biometry, Informatics and Epidemiology, University Hospital Bonn, Bonn, Germany.; 4 Institute of Pathology, Faculty of Medicine and University Hospital Cologne, University of Cologne, Cologne, Germany.; 5 Lutherian Hospital, Academic Teaching Hospital of the University Hospital Bonn, Bergisch Gladbach, Germany.; 6 Cancer Program, Broad Institute of MIT and Harvard, Cambridge, Massachusetts.; 7 Center for Cancer Research, Massachusetts General Hospital, Harvard Medical School Teaching Hospital, Charlestown, Massachusetts.; 8 Center of Functional Genomics, Berlin Institute of Health (BIH) at Charité – Universitätsmedizin Berlin, Berlin, Germany.; 9 Department of Hematology, Oncology and Cancer Immunology, Charité – Universitätsmedizin Berlin, corporate member of Freie Universität Berlin and Humboldt-Universität zu Berlin, Berlin, Germany.; 10 German Cancer Consortium (DKTK), Partner Site Berlin, and German Cancer Research Center (DKFZ), Heidelberg, Germany.

## Abstract

The incidence rates of vulvar squamous cell cancer (VSCC) have increased over the past decades, requiring personalized oncologic approaches. Currently, lymph node involvement is a key factor in determining prognosis and treatment options. However, there is a need for additional immune-related biomarkers to provide more precise treatment and prognostic information. Here, we used IHC and expression data to characterize immune cells and their spatial distribution in VSCC. Hierarchical clustering analysis identified distinct immune subtypes, of which the macrophage-rich subtype was associated with adverse outcome. This is consistent with our findings of increased lymphogenesis, lymphatic invasion, and lymph node involvement associated with high macrophage infiltration. Further *in vitro* studies showed that VSCC-associated macrophages expressed VEGF-A and subsequently induced VEGF-A in the VSCC cell line A-431, providing experimental support for a pro-lymphangiogenic role of macrophages in VSCC. Taken together, immune profiling in VSCC revealed tumor processes, identified a subset of patients with adverse outcome, and provided a valuable biomarker for risk stratification and therapeutic decision making for anti-VEGF treatment, ultimately contributing to the advancement of precision medicine in VSCC.

**Significance:**

Immunoprofiling in VSCC reveals subtypes with distinct clinical and biological behavior. Of these, the macrophage-rich VSCC subtype is characterized by poor clinical outcome and increased VEGF-A expression, providing a biomarker for risk stratification and therapeutic sensitivity.

## Introduction

Vulvar cancer accounts for less than 1% of malignancies worldwide, with approximately 59% of cases occurring in high-income countries (1, 2). Among the different histologic entities, vulvar squamous cell carcinoma (VSCC) accounts for 95% of cases (2). VSCC can be further classified into two subtypes based on their etiology: (i) tumors driven by high-risk human papillomaviruses (HPV-16, -18, -31, -33, and -45), responsible for 25% of VSCCs worldwide and 76% of tumors in women < 64 years of age (3); and (ii) tumors associated with chronic vulvar dermatoses, such as *lichen sclerosus*, which are primarily diagnosed in older patients and are associated with a worse prognosis (4).

The current standard of care for VSCC is radical surgery and, when appropriate, inguinal femoral lymphadenectomy with radiotherapy (5). This treatment approach has demonstrated excellent cure rates for early-stage tumors. However, the management of locally advanced, metastatic, and recurrent disease remains a challenge. In addition, while the HPV vaccination program provides protection against HPV-associated VSCC, HPV-independent and more aggressive forms of the disease continue to have a significant impact on public health and societal costs. In fact, the incidence of VSCC has steadily increased by 20% over the past two decades, with further escalation predicted because of the aging population (6, 7).

Currently, lymph node involvement serves as the single most important predictor of VSCC outcome and plays a critical role in treatment decisions (8). Tumor-associated macrophages (TAM) have been implicated in stimulating lymphogenesis through paracrine and/or cell autonomous modes and facilitating cancer cell invasion into the lymphatic circulation (9–11). Macrophages constitute a heterogeneous cell population with high plasticity and diverse effector functions (12, 13). They include classically activated proinflammatory M1 macrophages, alternatively activated anti-inflammatory M2 macrophages, and TAMs, which can exhibit properties of both subtypes. Antigens are valuable for the specific identification of TAMs, without necessarily characterizing a particular polarization status. One such marker is CD163, a myeloid cell–restricted hemoglobin/haptoglobin scavenger receptor, that has been shown to correlate with the pro-lymphangiogenic properties of TAMs (9).

However, the immune cell landscape in VSCC and the role of CD163^+^ TAMs within it remain poorly understood (14). In this study, we performed a comprehensive evaluation of immune cell populations and their spatial location in VSCC. Through immune profiling, we identified distinct subtypes and further experimentally elucidated the pro-lymphogenic potential of TAMs. In conclusion, our findings highlight stromal macrophages as a biomarker that can be readily applied in clinical practice and has the potential to aid in prognostic assessment and guide treatment decisions for patients with VSCC.

## Materials and Methods

### Patients and Specimens

The study population consisted of a retrospective sample set (archived formalin-fixed paraffin-embedded tissue) of 49 patients with primary VSCC diagnosed at the University of Bonn (Bonn, Germany) between 2002 and 2009 and treated according to the S2k guideline of the German Cancer Society and the German Society for Gynecology and Obstetrics. Only patients who underwent lymph node dissection were included in the study to provide a robust endpoint for lymphatic involvement; neoadjuvantly treated cases were excluded. Baseline characteristics were obtained from medical records; follow-up data were updated through March 2022. Histopathologic diagnosis was made according to World Health Organization criteria. The International Federation of Gynecology and Obstetrics (FIGO) system was used to assign tumor stages and grades (15). The Union for International Cancer Control tumor–node–metastasis (TNM) classification was used to assign tumor stages (16). The study was approved by the Institutional Review Board of the Medical Faculty of the University of Bonn, Bonn, Germany (228/15); written informed consent was obtained. Research was conducted in accordance with the Declaration of Helsinki.

### IHC

Staining of CD163, Foxp3, CD31, and D2-40 was performed on serial 4 µm sections from 49 patients using an automated staining system (DAKO TechMate 500; DAKO) and the streptavidin-biotin-peroxidase/DAB technique (DAKO) for visualization. In addition, we used results from tissue microarrays (TMA) of a previous study (17) performed on a subset of samples from the same subjects for CD3 (*n* = 41), CD20 (*n* = 43), CD68 (*n* = 42), Ki-67 (*n* = 40), and p53 (*n* = 41). Staining of p16^INK4a^ was performed on both serial sections and TMAs for all 49 patients. IHC antibodies are summarized in Supplementary Table S1.

### Assessment of Immunoreactions

Samples were analyzed masked to clinical details. Immunostaining was measured using a Leica DM LB2 microscope (Leica Microsystems) and the Pannoramic Viewer (3DHISTECH) or an Axio Observer D1 microscope (Zeiss). We quantified immune cell populations separately in two locations: (i) within the tumor mass (intratumoral); and (ii) within the peritumoral stromal compartment (stromal). Using whole tissue sections, three high-power fields (HPF) were identified with the highest immune cell infiltration in the tumor and the stroma, respectively. The fraction (%) of tumor (intratumoral analysis) and stroma (stromal analysis) in each HPF (0.28 mm^2^) was assessed. Specimens were then manually analyzed for Foxp3, p16^INK4a^, CD31, and D2-40 immunoreactivity. Quantification is presented as the mean number of immune cells normalized to the fraction of tumor (intratumoral analysis) and stroma (stromal analysis), respectively. CD163 immunoreactivity was quantified digitally using the AxioVision 4.7 image analysis software (Zeiss) according to previous studies (9, 18). Briefly, CD163 staining was calculated by measuring the number of pixels in the images after thresholding the stained images, and the immunoreactivity was then adjusted for the fraction of tumor (intratumoral analysis) and stroma (stromal analysis).

Whole tissue sections were also analyzed for the presence of lymphovascular invasion (LVI) and blood vessel invasion (BVI) using the criterion of cancer cell clusters within D2-40^+^ lymphovascular and CD31^+^ vascular spaces. To assess lymph vessel density (LVD) and blood vessel density (BVD), three stromal areas of highest neovascularization at the tumor invasion front (“hotspots”) were identified. The proportion (%) of stroma in each HPF was assessed; D2-40^+^ and CD31^+^ vessels were then counted and normalized to the extent of stroma in each HPF. Results are given as the mean number of vessels normalized to the proportion of stromal tissue.

### Identification of VSCC Etiologies

We used a panel of markers to identify the two etiology-based subtypes of VSCC. Specifically, we tested for the presence of high-risk HPV and performed staining for p53, p16^INK4a^ (a marker of HPV-transforming activity), and Ki-67 (19–21). The following three cutoffs were used: (i) p53 was considered positive if nuclear staining was present in >25% of cells; (ii) p16^INK4a^ was considered positive if there was a continuous strong nuclear plus cytoplasmic labeling of the basal and parabasal cells with upward extension involving ≥25% to 30% of the epithelial thickness (19); and (iii) Ki-67 labeling in ≥30% of the epithelium was used as a threshold to classify tumors into high and low reactivity (17). VSCCs associated with chronic vulvar skin disease (HPV-independent) were defined as high-risk HPV^neg^ and/or p16^INK4a neg^ and p53^pos^ and/or Ki-67^low^. VSCCs caused by HPV infection (HPV-associated) were defined as high-risk HPV^pos^ and/or p16^INK4a pos^ and p53^neg^ and/or Ki-67^high^.

### Cell Lines

Two human HPV-negative VSCC lines were used: A-431 (22, 23), obtained from Cell Lines Service (CLS), and CAL-39 (24), obtained from the German Collection of Microorganisms and Cell Cultures (DSMZ). Cells were cultured in DMEM (Gibco) supplemented with 10% FCS at 37°C in a humidified 5% CO_2_ atmosphere under strict endotoxin-free conditions. Cell line identity was verified by flow cytometry at regular intervals. Cells were tested for *Mycoplasma* by the PlasmoTest Mycoplasma Detection Kit (InvivoGen). Cell-free conditioned media was obtained from high-density cancer cell cultures at A-431 cell line passage numbers 21–44, and CAL-39 cell line passage numbers 1–2.

### Generation of Monocyte-derived Macrophages and *In Vitro* Polarized TAMs

Peripheral blood mononuclear cells (PBMC) were isolated from buffy coats of healthy donors by Ficoll-Paque (Biochrom) density centrifugation. The local ethics committee of the University of Bonn approved the human PBMC study protocol. Monocytes were enriched using the CD14 kit (Miltenyi Biotec). A purity of more than 98% was achieved, as assessed by flow cytometry. Monocytes were cultured at a density of 1.4 × 10^6^/mL in RPMI1640 (Gibco) supplemented with 10% FCS for 5 days. The lineage-determining GMCSF (50 U/mL or 20 ng/mL; ImmunoTools) was added for macrophage maturation. Cells were either left as untreated (monocyte-derived macrophages) or the following additional stimuli were added: (i) TAMs were induced by a 5-day incubation with cell-free supernatant from the VSCC cell cultures (40%, *in vitro* polarized TAMs); and (ii) M1 macrophages were induced by adding 20 ng/mL IFNγ (ImmunoTools) for 5 days.

### Coculture Conditions

In coculture experiments, *in vitro* polarized TAMs and A-431 cells were cultured either alone or together in a 2:1 ratio in the presence of 100 ng/mL lipopolysaccharide (LPS; Sigma-Aldrich) for 24 hours. Brefeldin A (Sigma-Aldrich), an inhibitor of intracellular protein transport, was added during the last 4 hours of coculture experiments to facilitate intracellular staining of VEGF-A.

### Flow Cytometry

Data were obtained on an LSR II flow cytometer (BD Biosciences), evaluating at least 10,000 events per sample by gating on live cells after excluding debris and doublets. To exclude dead cells from flow cytometric analysis, Hoechst 33258 (Sigma-Aldrich) was used for cell surface staining. Antibodies for flow cytometry are listed in Supplementary Table S2; fluorescence characteristics of tandem dye conjugates were checked periodically. VEGF-A was detected intracellularly using the Cytofix/Cytoperm kit (BD Biosciences); all other antigens were analyzed by cell surface staining. Analysis was performed using FlowJo software (TreeStar).

Monocyte-derived macrophages have been shown to exhibit increased autofluorescence (25), which may interfere with data interpretation (26). Accordingly, we used three rigorous methods to assess the expression of the antigens of interest. First, due to high levels of Fc receptors on macrophages, FcR Blocking Reagent (Miltenyi Biotec) was used to reduce nonspecific binding of antibodies. Second, similar cell numbers were acquired for the evaluation of target epitopes and negative controls; in coculture experiments, cell populations of interest were identified by their expression of phenotypic markers (EpCAM for cancer cells, CD14 for macrophages) and cell numbers were collected within these preselected positively stained populations. Third, isotype controls have been shown to be insufficient for differentiating positive from negative antigen expression, especially with respect to intracellular staining (27). Therefore, to assess the level of background staining for all target epitopes of interest, we used all fluorochromes of a given panel except the one being measured [fluorescence minus one (FMO) control]. In some experiments, we also used the corresponding isotype as a control. This was done separately for each cell type to account for cell type–specific autofluorescence. In the next step, the geometric mean fluorescence intensities (gMFI) of the FMO control samples were subtracted from the gMFIs of the experimental samples. The results are given as differential geometric mean fluorescence intensities (d-gMFI).

To compare the expression of VEGF in different cell types, cocultured *in vitro* polarized TAMs and A-431 cells were normalized to blood lymphocytes, which have been shown to be VEGF-A negative (28). For this approach, lymphocytes were isolated from buffy coats of healthy donors using lymphocyte separation medium (PAA Laboratories).

### Measurement of Cytokines

Cell-free supernatant was collected from high-density cancer cell cultures and after 5 days of culturing monocyte-derived macrophages or *in vitro* polarized TAMs. Signaling molecules IL1β, IL8, IL10, CXCL10 (IP-10), and TNFα were quantified by ELISA according to the manufacturer's instructions (BD Biosciences). Background cytokines/chemokines were subtracted from the *in vitro* polarized TAMs using the quantities detected in the conditioned media.

### Immunofluorescence Analysis

Intracellular staining of VEGF-A was performed using the Cytofix/Cytoperm kit (BD Biosciences). FcR Blocking Reagent (Miltenyi Biotec) was used to reduce nonspecific binding. The reaction was visualized with a secondary antibody conjugated to Alexa Fluor 488. Antibodies are listed in Supplementary Table S2. The cell membrane was labeled with PKH26 and the nucleus with Hoechst 34580 according to the manufacturer's instructions (Sigma-Aldrich). After immunoreaction, cells were flatmounted with Cytoseal 60 (Thermo Fisher Scientific). Images were captured using an Axio Observer.D1 microscope and AxioVision Rel.4.7 software (Zeiss). Negative controls without the primary antibody showed no significant fluorescence signal.

### Gene Expression Data Analysis

We retrieved the following uniformly processed Affymetrix microarray expression datasets from refine.bio on September 23, 2021: six VSCC cases [GSE63678 (29)], nine vulvar intraepithelial neoplasia (VIN) specimens [GSE5563 (30)], three lymph nodes with VSCC metastases [GSE28442 (31)], and 21 normal vulvar tissue samples [GSE63678, GSE5563, GSE28442]. Data were log_2_-transformed and quantile normalized; Z-score normalization was used to compare expression levels across microarray datasets.

We also obtained uniformly processed RNA-sequencing (RNA-seq) expression data from 12 HPV-positive primary VSCC samples [GSE183454 (32)] from GREIN (33) on May 13, 2023. Counts per million were normalized using the trimmed mean of M-values.

We used CIBERSORT (34) [through TIMER2.0 (35)] to computationally quantify immune cell fractions based on the expression data. The estimates provided reflect the absolute abundance of immune cells, which was determined by calculating the median expression of signature-related genes divided by the median expression of all genes.

### Clustering Analysis

Cell counts were log-transformed and Z-score normalized. Unsupervised hierarchical clustering of T cells and all immune cells was performed on the basis of Euclidean distance. CD3 clusters were categorized as follows: (i) “hot” for high CD3^+^ T-cell counts in the tumor and/or stroma; (ii) “cold” for low CD3^+^ T-cell counts in the tumor; and (iii) “immune excluded” for absent CD3^+^ T cells in the tumor and high CD3^+^ T-cell counts in the stroma. TAM clusters were categorized as follows: (i) “T/B cell^high^” or “immune activated” for high CD3^+^ T and/or CD20^+^ B-cell counts in tumor/stroma; (ii) “TAM^low^, T/B cell^low^” or “immune desert” for low immune cell counts in tumor/stroma; and (iii) “TAM^high^” for high CD163^+^ TAM counts in tumor/stroma.

### Statistical Analysis

We calculated the mean from multiple immunohistochemically determined immune cell and vessel counts for each patient. Values representing the entire cohort are presented as medians. The median immunostaining value was used as a threshold to categorize tumors into high and low reactivity groups for CD3, CD20, CD68, CD163, and Foxp3. In addition, the median number of blood and lymphatic vessels was used as a cutoff to divide cases into high and low reactivity groups. Poor differentiation [i.e., grades 2, 3 (36)] and advanced tumor stages [i.e., TNM pT2, 3 and FIGO II, III and IV (37)] have been shown to be associated with poor prognosis. Accordingly, cutoff points have been established between grades 2/3 and 1, as well as between TNM stages pT2/3 and pT1, and between FIGO stages II/III/IV and I.

Statistical analysis and visualization were performed using “R” version 4.1.1 in an R studio environment (The R Foundation for Statistical Computing). The *F* test was used to check the assumption of equal variances. The following specific “R” packages were used in our analysis: beeswarm, corrplot, survival, pheatmap. Two-sided Fisher exact tests and two-sided Student *t* tests or two-sided Mann–Whitney *U* tests were used to determine significance, as indicated (significance set at *P* value ≤ 0.05). Multiple hypothesis testing was performed using the Benjamini and Hochberg method (38), with final *P* values converted to FDR *q*-values (significance set at *q*-value ≤ 0.1). Pearson correlation coefficients were calculated to assess correlations. Values are presented as mean ± SEM or median ± median absolute deviation (MAD). Survival analysis was performed using the Kaplan–Meier method and curves were compared using the log-rank test. Multivariate survival analysis was performed using Cox proportional hazards regression model.

### Data Availability Statement

Affymetrix expression data are publicly available through Gene Expression Omnibus (GEO; GSE63678, GSE28442, and GSE5563) and were accessed at refine.bio (https://www.refine.bio/compendia?c=rna-seq-sample). RNA-seq data are publicly available via GEO (GSE183454) and were accessed at GREIN (http://www.ilincs.org/apps/grein/). CIBERSORT immune estimates were evaluated using TIMER2.0 (http://timer.cistrome.org). Data from previous studies were also used in the analysis in this article and these studies are referenced in the Materials and Methods section. Data from this study are available upon request. Software packages are published and available to the public; sources are cited in the Materials and Methods section.

## Results

### CD163^+^ TAMs are Present in Tumor and Peritumoral Stroma of VSCC

**FIGURE 1:**
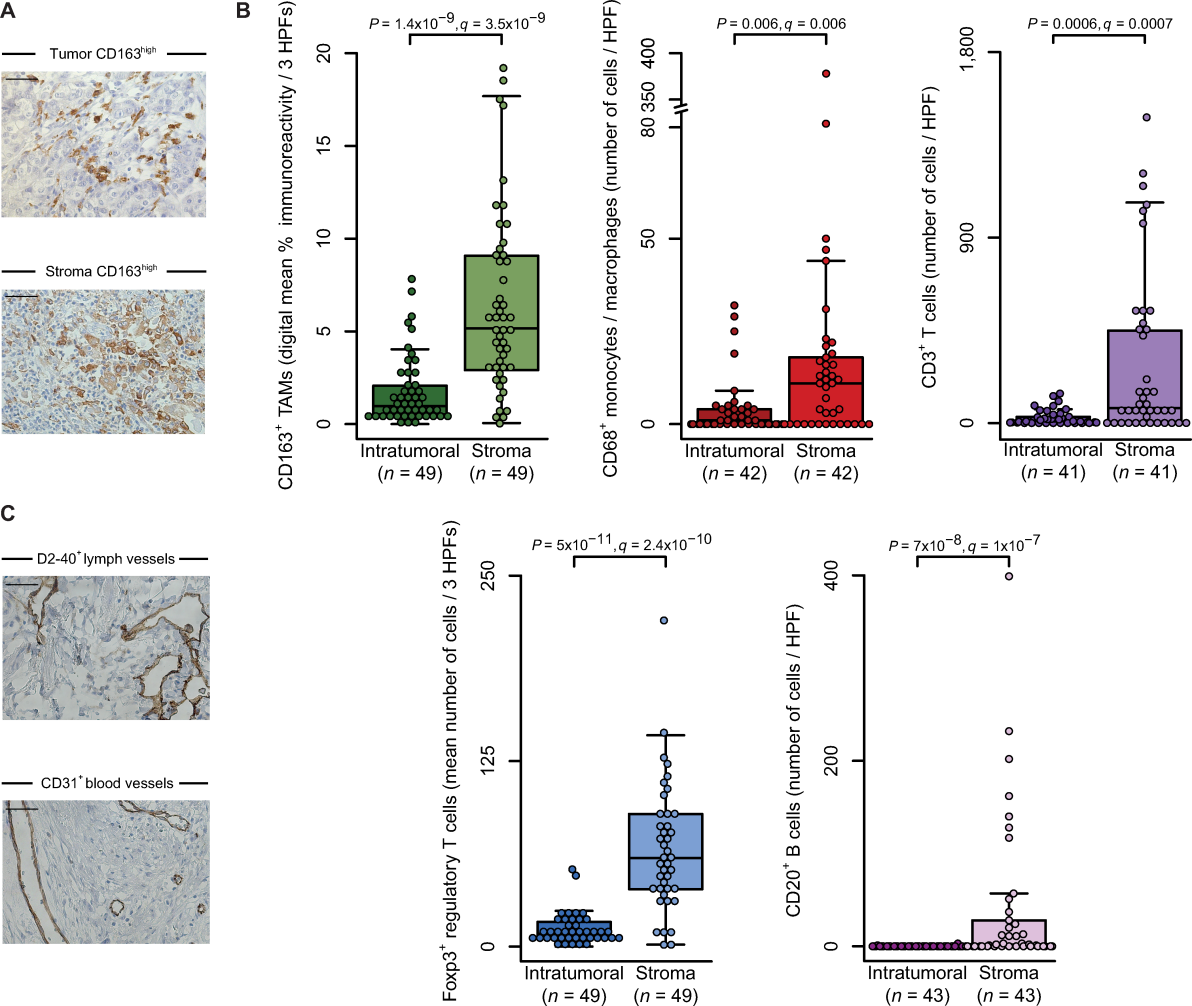
CD163^+^ TAMs are abundant in VSCC. **A,** Representative images show high CD163 expression in TAMs (brown cytoplasm/cell membrane) in tumor and stroma as visualized by IHC; hematoxylin (blue) was used for nuclear staining (bright field, 400 × magnification; scale bar length 50 µm). **B,** CD163 immunoreactivity was digitally recorded and analyzed separately in tumor and stroma using three HPFs; results are shown as mean immunoreactivity per tissue compartment in percent. CD68^+^, CD3^+^, Foxp3^+^, and CD20^+^ immune cell counts were determined in relation to stroma and tumor, respectively. Individual datapoints, shown as dots, overlap summary statistics boxplots with medians represented by horizontal center lines; a split axis is used for CD68 immunoreactivity. Significance analysis by two-sided Mann–Whitney U test with Benjamini–Hochberg procedure. **C,** Representative images of D2-40–stained lymphatic and CD31-stained vascular endothelium in the peritumoral stroma (brown; bright field, 400 × magnification; scale bar length 50 µm).

We used clinical specimens from patients with untreated HPV-positive and -negative VSCC (see Supplementary Table S3 for clinicopathologic characteristics of the discovery cohort) and directly analyzed CD163 protein expression by IHC. CD163^+^ TAMs were present in all specimens analyzed, albeit to varying degrees (median percent immunoreactivity ± MAD, 6 ± 4.7; range, 0.2–26; Supplementary Table S4). When densities were further assessed independently in the peritumoral and intratumoral regions, we found that TAMs were localized in both spatially defined tissue areas (Fig. 1A). However, the number of CD163^+^ TAMs differed significantly between spatial compartments (*q* = 3.5 × 10^−9^; Benjamini–Hochberg corrected Mann–Whitney *U* test; Fig. 1B) with a 5.3-fold higher median count in the peritumoral stroma (5.16 ± 4.29) compared with the tumor islets (0.97 ± 0.76).

In the next step, we characterized the VSCC-specific immune infiltrate more broadly. Similar to TAMs, Foxp3^+^ regulatory T cells were present in all samples (median number ± MAD, 66 ± 40; range, 1.3–2 67). In contrast, CD68^+^ monocytes/macrophages were detected in 69% (29 of 42), CD3^+^ T cells in 78% (32 of 41), and CD20^+^ B cells in 58% (25 of 43) of cases. Again, all immune cell types were particularly dense in the peritumoral stroma and significantly higher than in the tumor islets (all *q* < 0.006; Fig. 1B; Supplementary Table S4). Furthermore, we observed the presence of vascular processes in the peritumoral stroma (Fig. 1C). This was demonstrated by the expression of two markers: (i) D2-40 (39), which identifies the membrane protein podoplanin on the lymphatic endothelium; and (ii) the pan-vascular marker CD31.

### Immune Clusters Separate VSCC into Distinct Subgroups

**FIGURE 2:**
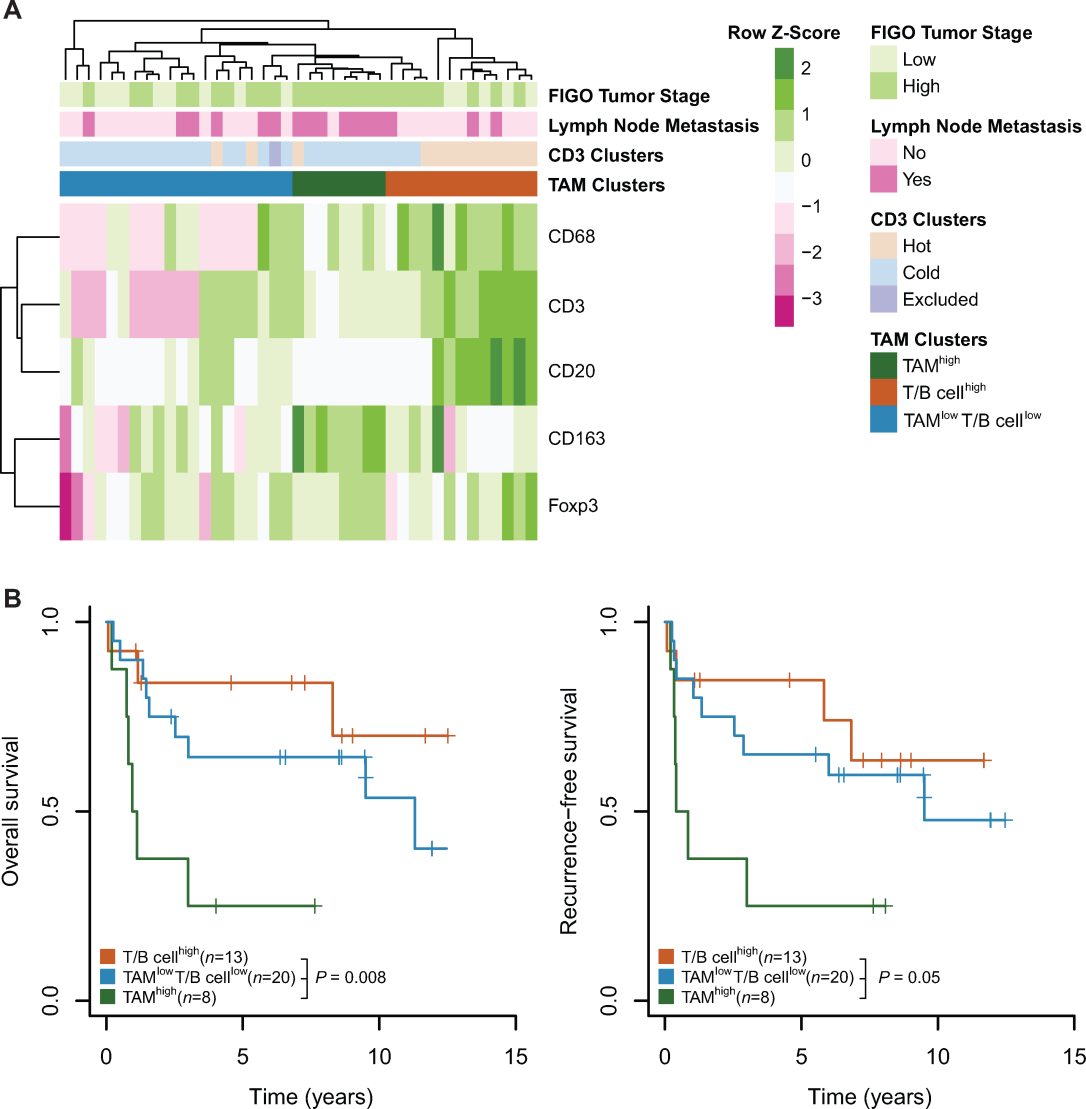
The TAM^high^ immune cell cluster is associated with poor outcome. **A,** Heat map shows the distribution of immune cells (intratumoral and stromal combined). Immunoreactivity was recorded as in Fig. 1B. Unsupervised clustering of log_2_-transformed cell count data from 41 samples was performed using Euclidean distance. **B,** Kaplan–Meier plots depict OS and RFS of patients stratified by TAM clusters from **A**; significance analysis by log-rank test.

Next, we aimed to gain deeper insights into the role of TAMs and their significance in shaping the immune landscape of VSCC. To achieve this, we performed unsupervised hierarchical clustering of all immune cell populations, considering tumor and stroma compartments, in the 41 samples for which all immune cell types were characterized. This analysis identified three distinct immune cell clusters (Fig. 2A): one group of eight VSCC samples exhibited a pronounced TAM infiltrate (termed “TAM^high^”), another group of 13 VSCCs showed high CD3^+^ T- and/or CD20^+^ B-cell counts (“T/B cell^high^” or “immune activated”), while the third group of 20 VSCCs lacked significant immune cell presence (“TAM^low^, T/B cell^low^” or “immune desert”).

The inclusion of TAMs in the clustering analysis expanded the repertoire of immune phenotypes, traditionally based on CD3^+^ T-cell densities. Using unsupervised hierarchical clustering based solely on T-cell densities, we identified three immune cell clusters within our VSCC sample set (Supplementary Fig. S1A). Specifically, 13 VSCCs exhibited a “hot” immune phenotype, 27 VSCCs were characterized as “cold,” and one VSCC showed an “immune excluded” profile.

When superimposing the two clustering approaches (our novel approach incorporating TAMs and the traditional T cell–based approach), we observed significant overlap for two of the three clusters from our TAM-based approach. In particular, 10 of the 13 samples in the “immune activated” TAM cluster were also classified as T cell hot, whereas 17 of the 20 samples in the “immune desert” TAM cluster were also classified as T cell cold. The TAM^high^ immune phenotype emerged as a new cluster, consisting of one sample with pronounced T-cell infiltration (hot) and seven VSCCs with few T cells (cold). As this distinct subset suggests a potential difference in tumor biology, we next aimed to better understand whether the TAM^high^ cluster was indeed associated with unique tumor characteristics and features. VSCCs of the TAM^high^ immune cell cluster were characterized by a high tumor stage (8/8 vs. 18/33 in other subtypes; *P* = 0.02, Fisher exact test) and lymph node positivity (7/8 vs. 9/33; *P* = 0.003). Consistent with these observations, patients with TAM^high^ VSCC tumors had shorter overall survival (OS; *P* = 0.008, log-rank test) and recurrence-free survival (RFS; *P* = 0.05; Fig. 2B) compared with other groups, highlighting that the TAM^high^ subset correlates with a more aggressive tumor biology.

### Characterization of Immune Cell Topographies in VSCC

Given the implications of the TAM^high^ cluster on the immune landscape, we next aimed to elucidate the relationship between myeloid and lymphoid cells by leveraging spatial distributions. To this end, we performed a parallel analysis of immune cell infiltrations in two different tissue areas (intratumoral and stromal). Within the same tissue area, CD163^+^ TAMs showed significant spatial correlations exclusively with CD68^+^ monocytes/macrophages (tumor: *r* = 0.42, *P* = 6 × 10^−3^; stroma: *r* = 0.43, *P* = 4 × 10^−2^; Pearson; Supplementary Fig. S1B), confirming their subset classification as CD68^+^ cells. Across spatial compartments, CD163^+^ TAMs showed infiltration patterns with positive correlations (*r* = 0.66; *P* = 3 × 10^−6^), but no correlations with other immune cells were observed. These findings suggest that the immune cell populations may occupy distinct niches within the tumor microenvironment, resulting in limited spatial overlap and correlation with TAMs.

### CD163^+^ TAMs in the Stroma are Associated with Unfavorable Outcome

On the basis of our finding that the TAM^high^ VSCC immune cluster predicts adverse outcome, we further investigated the prognostic significance of TAMs and their spatial location to determine whether TAM density alone could be a biomarker for risk stratification and which tissue compartment plays a pivotal role.

**FIGURE 3:**
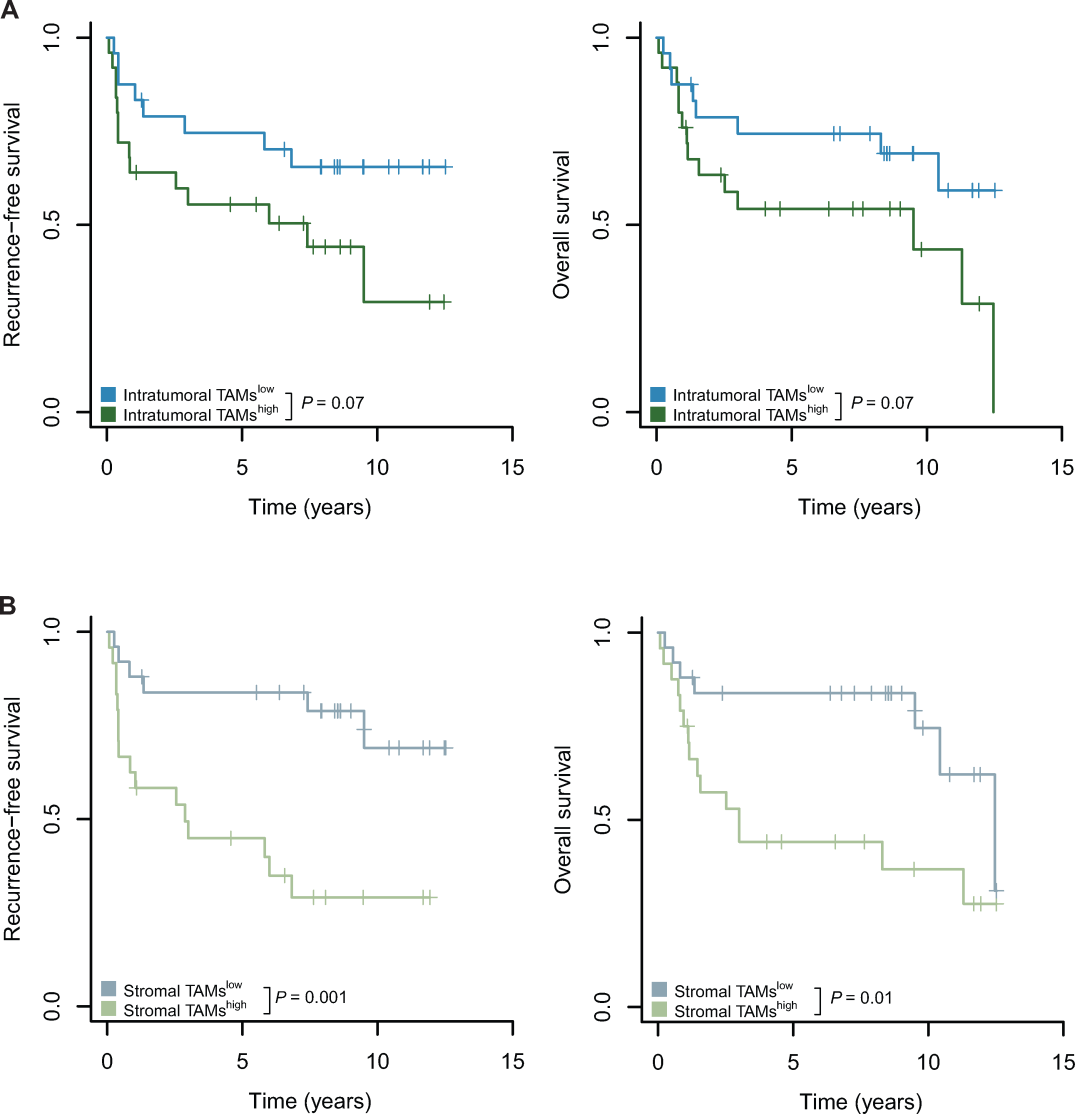
High numbers of stromal CD163^+^ TAMs are associated with adverse outcome. **A,** CD163 immunoreactivity was recorded intratumorally as in Fig. 1B; data are presented as mean immunoreactivity normalized to tissue compartments in percent. Cutoff values were determined using median CD163 expression to classify low-risk and high-risk groups; Kaplan–Meier curves are shown for each risk tier; log-rank test results are reported. **B,** CD163 immunoreactivity was recorded in the stroma as in A; Kaplan–Meier curves are shown for each risk tier; log-rank test results are reported.

Intratumoral CD163^+^ TAMs showed a trend in differences for RFS (*P* = 0.07, log-rank test) and OS (*P* = 0.07; Fig. 3A), although the results did not reach statistical significance. However, a high number of stromal CD163^+^ TAMs was significantly associated with poor RFS (*P* = 0.001) and OS (*P* = 0.01; Fig. 3B). The adverse prognostic impact of stromal CD163^+^ TAMs remained significant (RFS: *P* = 0.02, Wald test; Table 1) in a multivariate model when correcting for lymph node metastasis, a known clinical prognostic factor in VSCC (8). Women with tumors characterized by a high number of stromal TAMs had a HR for recurrence of 4.4 [95% confidence interval (CI), 1.69–11.35], suggesting that stromal CD163^+^ TAM density can serve as an independent unfavorable prognostic factor in VSCC. Importantly, our study used only primary VSCC specimens and found an association between stromal TAMs at initial diagnosis and subsequent adverse patient outcomes after a median follow-up of 6.79 years (Supplementary Table S3), highlighting the prognostic potential of TAMs in the context of VSCC.

**TABLE 1:** Density of stromal TAMs affect outcome. Survival analysis of intratumoral and stromal immune cell counts

	Recurrence-free survival					
	Univariate analysis			Multivariate analysis^a^		
Variable	HR	95% CI	*P*-value	HR	95% CI	*P*-value
Intratumoral CD163^+^ TAMs (high vs. low)	2.2	0.93–5.32	0.07			
Stromal CD163^+^ TAMs (high vs. low)	4.4	1.69–11.35	0.001	3.5	1.28–9.63	0.02
Lymph node involvement (present vs. absent)	2.9	1.26–6.86	0.01	1.89	0.77–4.62	0.2
	**Overall survival**					
	**Univariate analysis**			**Multivariate analysis** **^a^**		
**Variable**	**HR**	**95% CI**	***P*-value**	**HR**	**95% CI**	***P*-value**
Intratumoral CD163^+^ TAMs (high vs. low)	2.2	0.92–5.3	0.07			
Stromal CD163^+^ TAMs (high vs. low)	2.9	1.2–7.28	0.02	2.3	0.9–5.84	0.09
Lymph node involvement (present vs. absent)	3.7	1.57–8.57	0.003	2.95	1.2–7.1	0.02

Abbreviations: CI, confidence interval; HR, hazard ratio.

^a^Only variables significantly associated in the univariate analysis were included in the multivariate analysis.

### CD163^+^ TAMs are Indicative of an Aggressive VSCC Tumor Phenotype

Next, we aimed to gain insight into the unique tumor biology associated with TAM-enriched tumors by analyzing clinical risk factors. We observed a significant increase in the number of stromal CD163^+^ TAMs with higher tumor stages, regardless of whether TNM (*q* = 0.04, Benjamini–Hochberg corrected Fisher exact test) or FIGO classifications (*q* = 0.001) were used (Table 2). Furthermore, we found that stromal CD163^+^ TAMs showed a positive correlation with lymph node involvement (*q* = 0.1), consistent with our observation in the TAM^high^ immune cell cluster (Fig. 2A). To explore potential associations between CD163^+^ TAMs and VSCC etiologic pathways (3, 4), we used a combination of markers including p53, Ki-67, high-risk HPV, and p16^INK4a^ to identify relevant subgroups. However, our analysis showed no significant difference in CD163^+^ TAMs between viral and nonviral etiologies (all *P* > 0.4; Supplementary Table S5). In addition, none of the other immune cell types showed significant correlations with clinicopathologic variables (all *q* > 0.1; Table 2). Of note, no association was observed between CD68 expression (a general marker for myeloid cells) and clinical indicators, suggesting that CD163 serves as a more specific marker for TAMs in VSCC.

**TABLE 2:** Stromal TAMs indicate aggressive tumor biology. Association between intratumoral and stromal immune cell counts and clinicopathologic parameters

		Intratumoral				Stromal			
Variable		High	Low	*P*-value	*q*-value	High	Low	*P*-value	*q*-value
**CD163^+^ TAMs (*n* = 49)**									
Tumor stage (TNM)	pT2, pT3	15	12	0.57	1	19	8	0.0014	0.04
	pT1	10	12			5	17		
Tumor stage (FIGO)	II, III, IV	18	12	0.15	1	22	8	0.00002	0.001
	I	7	12			2	17		
Tumor grade	2, 3	25	20	0.05	0.63	22	23	1	1
	1	0	4			2	2		
Lymph node involvement	Present	12	5	0.07	0.7	13	4	0.007	0.1
	Absent	13	19			11	21		
Metastasis	Present	2	1	1	1	2	1	0.61	1
	Absent	23	23			22	24		
**CD3^+^ T cells (*n* = 41)**									
Tumor stage (TNM)	pT2, pT3	11	12	0.76	1	10	13	0.35	1
	pT1	10	8			11	7		
Tumor stage (FIGO)	II, III, IV	13	13	1	1	13	13	1	1
	I	8	7			8	7		
Tumor grade	2, 3	19	18	1	1	18	19	0.61	1
	1	2	2			3	1		
Lymph node involvement	Present	7	9	0.53	1	7	9	0.53	1
	Absent	14	11			14	11		
Metastasis	Present	2	1	1	1	2	1	1	1
	Absent	19	19			19	19		
**CD20^+^ B cells (*n* = 43)**									
Tumor stage (TNM)	pT2, pT3	1	24	1	1	11	14	0.2	1
	pT1	1	17			12	6		
Tumor stage (FIGO)	II, III, IV	2	26	0.53	1	14	14	0.75	1
	I	0	15			9	6		
Tumor grade	2, 3	2	37	1	1	21	18	1	1
	1	0	4			2	2		
Lymph node involvement	Present	1	15	1	1	7	9	0.36	1
	Absent	1	26			16	11		
Metastasis	Present	0	3	1	1	1	18	0.59	1
	Absent	2	38			22	2		
**CD68^+^ macrophages (*n* = 42)**									
Tumor stage (TNM)	pT2, pT3	13	11	0.76	1	12	12	1	1
	pT1	8	10			9	9		
Tumor stage (FIGO)	II, III, IV	15	12	0.52	1	14	13	1	1
	I	6	9			7	8		
Tumor grade	2, 3	20	18	0.61	1	18	20	0.61	1
	1	1	3			3	1		
Lymph node involvement	Present	9	7	0.75	1	8	8	1	1
	Absent	12	14			13	13		
Metastasis	Present	2	1	1	1	2	1	1	1
	Absent	19	20			19	20		
**Foxp3^+^ T cells (*n* = 49)**									
Tumor stage (TNM)	pT2, pT3	14	13	0.78	1	14	13	1	1
	pT1	10	12			11	11		
Tumor stage (FIGO)	II, III, IV	16	14	0.56	1	17	13	0.39	1
	I	8	11			8	11		
Tumor grade	2, 3	23	22	0.61	1	22	23	0.61	1
	1	1	3			3	1		
Lymph node involvement	Present	11	6	0.14	1	10	7	0.55	1
	Absent	13	19			15	17		
Metastasis	Present	1	2	1	1	1	2	0.61	1
	Absent	23	23			24	22		

### CD163^+^ TAMs Contribute to Tumor Lymphogenesis

We next aimed to further investigate the observed association between TAMs and lymph node involvement. Lymph node metastasis is one of the most important prognostic factors in VSCC (8). The process involves several sequential steps, including vessel proliferation (reflected by vessel density) and the spread of tumor cells through lymphatic vessels to the lymph nodes (vascular invasion). Lymphatic vessels, delineated by D2-40, were observed in the peritumoral stroma of all VSCC samples, albeit at different densities, indicating different states of neolymphogenesis (median number ± MAD, 6.68 ± 4.33; range, 1.33–53; Supplementary Table S4). Similarly, varying degrees of stromal vascularization with CD31^+^ blood vessels were observed (13.38 ± 6.99; 4.2–54.74). Further evaluation of stained vessels for tumor cell spread identified LVI in 28/49 (57%) and BVI in 5/49 (10%) cases (Supplementary Table S4).

**FIGURE 4:**
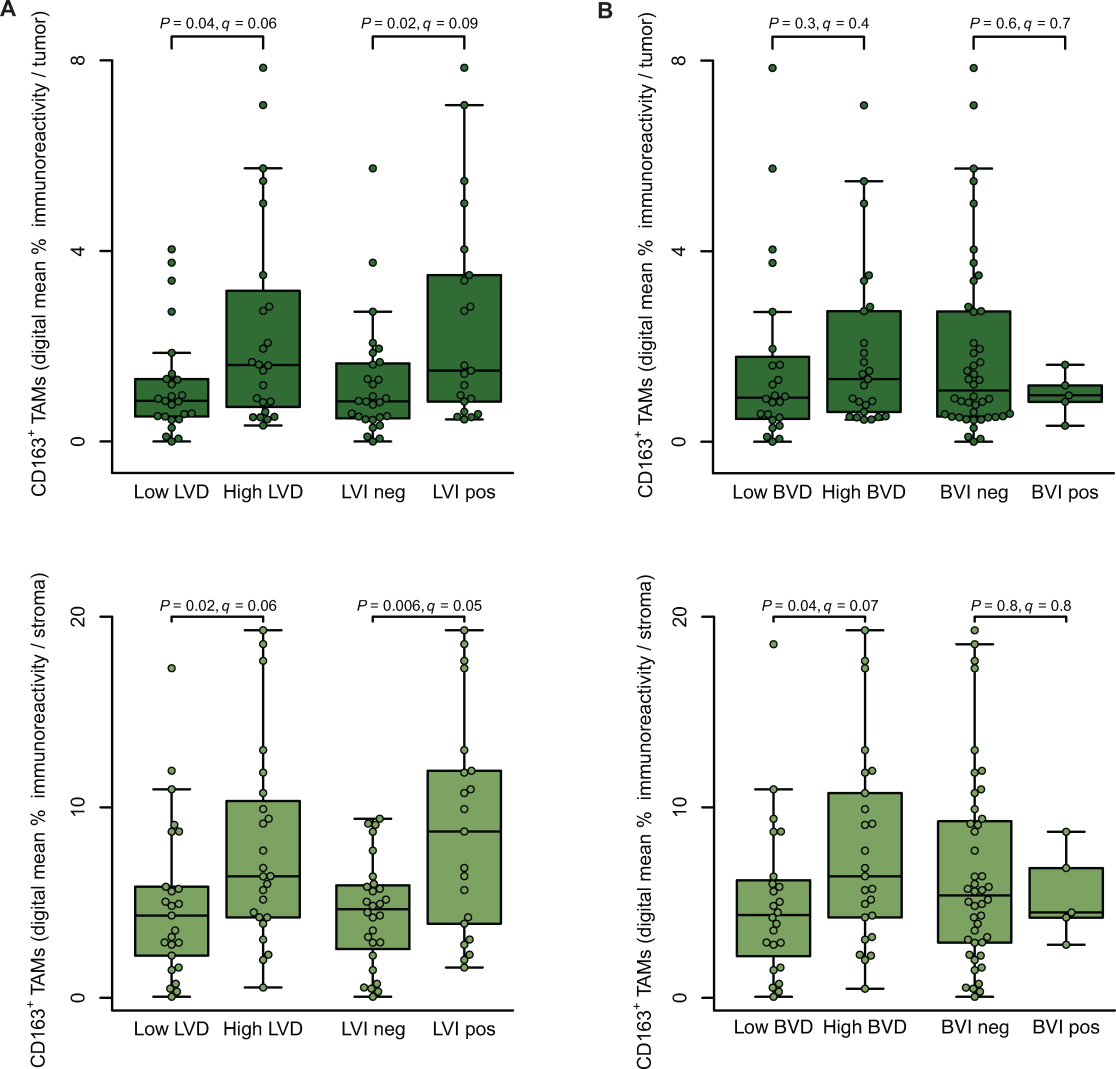
CD163^+^ TAMs are associated with lymphogenesis. **A,** LVD and LVI were assessed in the stroma using D2-40–stained lymphatic endothelium. Intratumoral and stromal CD163 immunoreactivity was recorded as in Fig. 1B; data are presented as mean immunoreactivity normalized to tissue compartments in percent. CD163^+^ TAMs were categorized on the basis of high/low LVD and the presence/absence of LVI. Individual datapoints, shown as dots, overlap summary statistics boxplots with medians represented by horizontal center lines. Significance analysis by two-sided Mann–Whitney U test with Benjamini–Hochberg procedure. **B,** BVD and BVI were assessed in the stroma using CD31-stained vessels. Intratumoral and stromal CD163 immunoreactivity was recorded as in Fig. 1B; data are presented as mean immunoreactivity normalized to tissue compartments in percent. CD163^+^ TAMs were categorized on the basis of high/low BVD and the presence/absence of BVI. Individual datapoints, shown as dots, overlap summary statistics boxplots with medians represented by horizontal center lines. Significance analysis by two-sided Mann–Whitney U test with Benjamini–Hochberg procedure.

Our subsequent correlation analysis revealed that increased numbers of CD163^+^ TAMs were associated with higher LVD, regardless of their tissue compartment (tumor and stroma: *q* = 0.06; Benjamini–Hochberg corrected Mann–Whitney U test; Fig. 4A). Moreover, CD163^+^ TAMs were positively correlated with LVI, again independent of their spatial location (tumor: *q* = 0.09; stroma: *q* = 0.06). In contrast, when evaluating hemangiogenesis, only stromal CD163^+^ TAMs were associated with increased BVD (*q* = 0.07; Fig. 4B). Together, these results suggest that TAMs, irrespective of their location, are associated with lymphogenesis in VSCC, potentially facilitating lymphatic tumor invasion and metastasis.

### Independent Cohorts Validate a Pro-lymphangiogenic Role of CD163^+^ TAMs

We independently validated our findings using two separate VSCC sample sets of bulk RNA expression data (31, 32). First, to estimate immune cell composition from these expression data, we utilized support vector regression modeling (CIBERSORT), which deconvolutes cellular composition and calculates immune cell types based on gene expression patterns (34). This indirect computational approach has previously been applied to other cancer types with successful results (40). CIBERSORT uses expression profiles of pure cell type populations as a reference and thus estimates the proportion of immune cell types in bulk tissue based on the presence and absence of multiple cell type–specific markers. Consistent with our IHC cohort, we observed macrophage, T-cell, and B-cell phenotypes in both primary tumors and metastatic lymph nodes from patients with VSCC (Supplementary Fig. S2A).

We next validated the gene expression data using CIBERSORT-estimated immune cell infiltration, as markers can be expressed by different cell populations, potentially leading to ambiguous results. This allowed us to confirm the correlation between CD163 expression and macrophage infiltration in primary VSCC (*r* = 0.72; *P* = 0.008; Pearson; Supplementary Fig. S2B), demonstrating the reliability of CD163 as a marker to identify macrophages in this context. Using the RNA data, we observed that increased podoplanin expression, which reflects a high degree of lymphatic endothelial cells, was associated with higher CD163 levels in both primary VSCC (*P* = 0.03, Student *t* test; Supplementary Fig. S2C) and lymph nodes from patients with VSCC (*r* = 0.89; *P* = 0.007; Pearson; Supplementary Fig. S2D), supporting our IHC findings. In addition, we confirmed the correlation between CD163 expression and the lymphatic system by analyzing matched tumor-free and metastatic lymph nodes from the same patients. We found that CD163 expression was increased in the presence of VSCC (median 5 ± 0.2) compared with tumor-free lymph nodes (median 2 ± 0.5; Supplementary Fig. S2E).

### VSCC Induces a Macrophage Phenotype Switch

**FIGURE 5:**
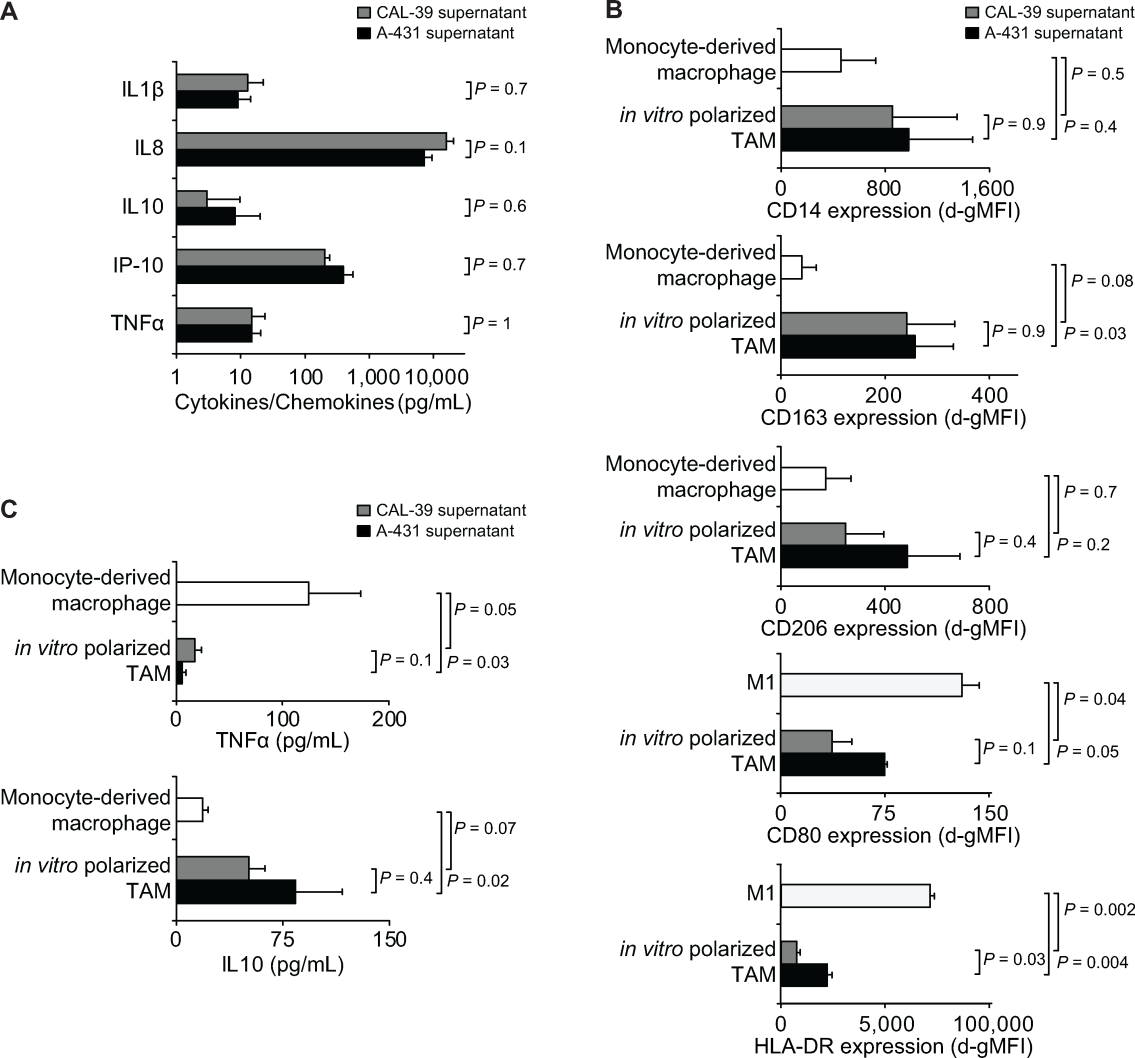
Characterization of *in vitro* polarized TAMs. **A,** Cytokines and chemokines detected in the supernatant of high-density VSCC cultures by ELISA. **B,** Healthy donor monocytes were differentiated into macrophages using GMCSF and exposed to different culture conditions: medium alone (monocyte-derived macrophage), VSCC supernatant (*in vitro* polarized TAM), or 20 ng/mL IFNγ (M1). Flow cytometry on day 5 determined surface antigen expression, presented as d-gMFIs. **C,** Macrophages were generated as in B; TNFα and IL10 levels were quantified in the supernatant by ELISA on day 5; background levels were subtracted. Data represent results from two individuals and/or experiments and are presented as mean ± SEM; significance was determined by two-sided Student *t* test.

To elucidate the underlying effects of TAMs on the lymphatic system in VSCC and to validate our findings from the correlation analyses, we next established an *in vitro* model of myeloid cells treated with conditioned media from the VSCC cell lines A-431 and CAL-39. Analysis of signaling molecules in the conditioned media showed a consistent pattern of cytokines and chemokines with comparable concentrations in both cell lines (all *P* > 0.5; Student *t* test; Fig. 5A).

Macrophages cultured in the presence of VSCC-conditioned media from both cell lines (*in vitro* polarized TAMs) showed comparable expression levels of CD14 compared with monocyte-derived macrophages cultured without VSCC-conditioned media, confirming the myeloid origin of both cell types (all *P* > 0.3; Fig. 5B). Of the two TAM-specific antigens analyzed, CD163 and CD206 (41), only CD163 showed increased expression in *in vitro* polarized TAMs compared to monocyte-derived macrophages, independent of the VSCC cell line (*P* = 0.03 and *P* = 0.08; Fig. 5B). Further examination of the cytokines of *in vitro* polarized TAMs revealed a decrease in TNFα (*P* = 0.03 and *P* = 0.05) and an increase in IL10 (*P* = 0.02 and *P* = 0.07; Fig. 5C). When contrasted with classically activated M1 macrophages, distinct expression patterns of costimulatory and activating antigens emerged (Fig. 5B). Specifically, *in vitro* polarized TAMs showed decreased expression of CD80 (*P* = 0.05 and *P* = 0.04) and HLA-DR (*P* = 0.004 and *P* = 0.002). Together, these findings suggest that *in vitro* polarized CD163^+^ TAMs exhibit a combination of proinflammatory and anti-inflammatory properties, making them a relevant model to recapitulate the *in vivo* scenario.

### TAMs are Associated with a Pro-lymphangiogenic Milieu Mediated by VEGF-A

VEGFs, including VEGF-A, VEGF-C, and VEGF-D, are a family of proteins that are key regulators of lymphogenesis (42) in normal and cancer tissues (43). To identify the VEGF protein most critical for lymphogenesis in VSCC, we analyzed gene expression data for VEGF-A, VEGF-C, and VEGF-D from the different stages in VSCC development: normal vulvar tissue, the precursor lesion VIN, and VSCC. Among the VEGF proteins analyzed, only VEGF-A was low in normal tissue (median, −0.52; range, −0.92 to 0.18), showed increased levels in the precursor lesion (median, 0.34; range, 0.042–0.66; vs. normal *q* = 2 × 10^−5^, Benjamini–Hochberg corrected two-sided Mann–Whitney U test) and had the highest levels in VSCC (median, 0.78; range, −0.26 to 4.9; vs. normal *q* = 0.002; Supplementary Fig. S3). Given this stepwise upregulation in VSCC development, we focused on VEGF-A to investigate the pro-lymphangiogenic properties of *in vitro* polarized TAMs.

**FIGURE 6:**
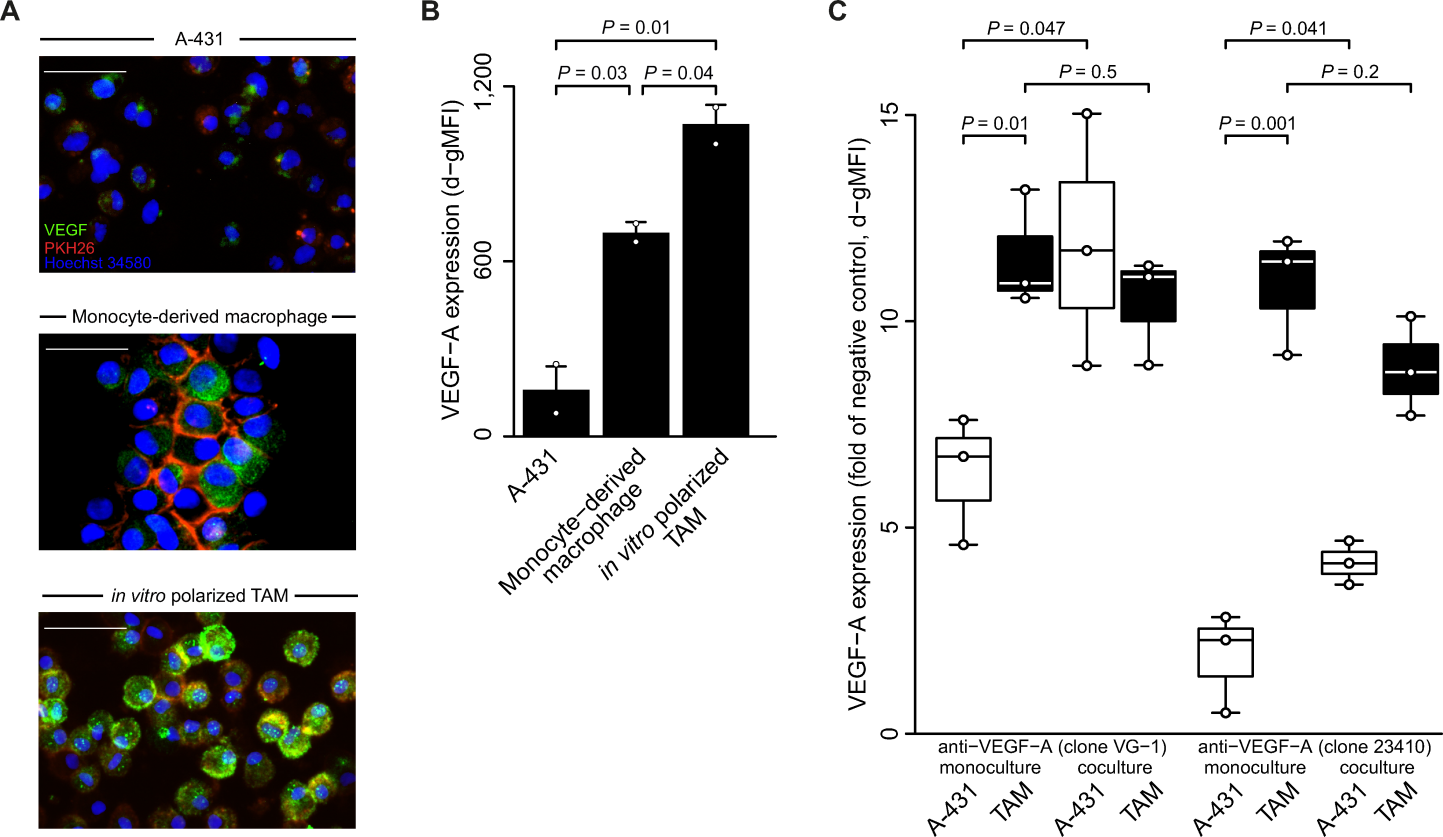
VEGF-A expression by *in vitro* polarized TAMs. **A,** Healthy donor monocytes were differentiated into macrophages using GMCSF and exposed to different culture conditions: medium alone (monocyte-derived macrophage) or A-431 supernatant (*in vitro* polarized TAM). Immunofluorescent staining was performed on day 5 and compared with A-431 cells. Cells were stained with anti-VEGF-A (clone VG-1; cytoplasma, green), PKH26 (cell membrane, red), and Hoechst 34580 (cell nucleus, blue). Representative overlay figures are depicted (32x magnification); white scale bar length 200 µm. **B,** Macrophages were generated as in A and intracellular VEGF-A expression was determined by flow cytometry (anti-VEGF-A clone 23410) on day 5. Data combine results from two independently analyzed individuals. Data are given as d-gMFIs. Individual datapoints, shown as dots, overlap summary statistics barplots (mean ± SEM). Significance analysis by two-sided Student *t* test. **C,** Macrophages were generated as in A, and *in vitro* polarized TAMs and A-431 cells were cultured alone (monoculture) or together (coculture with 2:1 ratio) in the presence of LPS. Intracellular VEGF-A expression was determined by flow cytometry using two anti-VEGF-A antibody clones as indicated after 24 hours of coculture following a 4-hour restimulation with Brefeldin A. For comparison between different cell types, specific cell populations of interest were identified on the basis of their expression of phenotypic markers (EpCAM for cancer cells, CD14 for macrophages), a negative control was included, and cocultured cells were normalized to VEGF expression of blood lymphocytes. Data combine results from three individuals and are given as d-gMFIs. Individual datapoints, shown as dots, overlap summary statistics boxplots with medians represented by horizontal center lines. Significance analysis by two-sided Student *t* test.

We first performed immunofluorescence to detect and localize VEGF-A expression. Using an antibody (clone VG-1) designed to recognize the most commonly expressed isoforms, VEGF-A_165_ and VEGF-A_121_, we identified a heterogeneous expression of VEGF-A throughout the cytoplasm in A-431 VSCC cells and monocyte-derived macrophages (Fig. 6A). After exposure to tumor conditioned media, a more intense cytoplasmic expression of VEGF was observed in *in vitro* polarized TAMs. Next, we quantified and formally assessed the difference by flow cytometry using a different antibody (clone 23410) to detect VEGF-A_165_ and VEGF-A_121_, ensuring the robustness of our results. Again, VEGF-A levels were highest in *in vitro* polarized TAMs compared with controls (vs. monocyte-derived macrophages *P* = 0.04; vs. A-431 *P* = 0.01; Student *t* test; Fig. 6B).

Finally, we investigated the potential reciprocal effects between VSCC cells and *in vitro* polarized TAMs, which also under these conditions showed high IL10 and low TNFα expression, consistent with our previous findings (Fig. 5C). This experiment was performed with both anti-VEGF-A antibodies (clones VG-1 and 23410). Again, higher levels of VEGF-A were observed in *in vitro* polarized TAMs compared with VSCC cells, in both monoculture settings (all *P* < 0.01; Fig. 6C). Interestingly, coculture of VSCC cells with *in vitro* polarized TAMs increased VEGF-A levels in VSCC compared with VSCC cells alone (all *P* < 0.05). In contrast, the total secretion of VEGF-A by TAMs in coculture remained unchanged compared with monocultured TAMs (all *P* > 0.2). We evaluated these effects by flow cytometry in individual cell populations using cell type–specific markers, allowing us to precisely assess the impact of coculture on VEGF-A expression in specific cell types. Together, our exploratory findings support the notion that CD163^+^ TAMs play a critical role in orchestrating lymphogenesis in VSCC by exhibiting enhanced VEGF-A expression and triggering increased VEGF-A levels in VSCC tumor cells.

## Discussion

This study provides insights into the involvement of CD163^+^ TAMs in the development and progression of VSCC. Our findings highlight the critical role of CD163^+^ TAMs as a prognostic factor in VSCC, underscoring the clinical relevance of TAMs and their potential impact on the management and treatment of patients with VSCC.

Our analysis identified a TAM^high^ immune cell cluster indicative of tumor aggressiveness, extending previous observations in other cancer types (44). More specifically, our results highlight that TAMs alone are associated with poor outcome, suggesting that TAMs are indeed the drivers in this immune cell cluster. Furthermore, the spatial distribution of TAMs affects tumor growth and progression, suggesting that the activity of TAMs is modulated by microlocalization (45). To date, only one previous study has investigated TAMs in VSCC (14). In our study, we more than doubled the number of HPV-positive VSCCs examined in the previous study (*n* = 21) and included cases with HPV-dependent and HPV-independent etiologies. Similar to our results, the previous study also reported a higher number of CD163^+^ macrophages in the stroma compared with the tumor but did not further evaluate this as a predictive factor. Nevertheless, consistent with our findings, studies in other tumor types have shown that TAMs located in the stroma, but not in the tumor islets, negatively affect outcome (46, 47).

In addition, we performed functional *in vitro* studies and discovered an underlying mechanism by which TAMs play a lymphogenic role in VSCC. Similar cancer-driving properties of TAMs have been reported in other cancer types (9–11), but the specific role of TAMs in VSCC has remained elusive. Our *in vitro* studies used conditioned media to induce polarization of myeloid cells into TAMs. Consistent with previous studies (48, 49), these *in vitro* polarized TAMs expressed CD163 but not CD206 and produced high levels of IL10, indicating that our model captures important biological features of TAMs. We found enhanced VEGF-A expression in *in vitro* polarized TAMs as well as in the cocultured A-431 VSCC cells, suggesting that TAM-related lymphogenesis may be responsible for the observed association between TAM density and lymph node involvement in VSCC. It is well established that VEGF plays a role in lymphogenesis (50). We used antibodies that detect the isoform VEGF-A_121_, which is known to promote vascular endothelial cell proliferation and angiogenesis, but also to stimulate lymphogenesis, at least in part through induction of the lymphangiogenic growth factor VEGF-C (51). Of note, in contrast to normal tissue, tumor cells were found to predominantly secrete VEGF-A_121_ (52). Our finding of further increased VEGF-A levels in VSCC cells cocultured with CD163^+^ TAMs is supported by the previous observation that TAMs can induce the p38 MAPK/NFκB/COX-2–dependent secretion of VEGF-A in basal cell cancer cells (53). Further support linking the predictive marker role of TAMs to our *in vitro* results comes from animal models in which the reduction of stromal TAMs altered the tumor microenvironment, resulting in a marked decrease in VEGF and suppression of cancer growth and metastasis (54).

A potential limitation of our study is the restriction to patients who underwent lymph node dissection. This led to an underrepresentation of early stage tumors: only two pT1a tumors received lymph node dissection due to a higher tumor stage at biopsy. However, our approach was motivated by the goal of using a clinically important and robust endpoint (i.e., histologically defined lymph node status).

In summary, this study expands the current repertoire of HPV-associated and HPV-independent VSCC subtypes by providing evidence for a TAM^high^ immune cell cluster with a distinct immune profile and a unique tumor biology. Although larger cohorts are needed to validate our pilot study and draw definitive conclusions, the consistency of our clinical and *in vitro* findings may serve as a foundation for future personalized treatment approaches. Reprogramming of TAMs limits tumor growth in mice (55), underscoring the need to advance TAM-targeted therapeutic approaches in humans. Anti-VEGF agents are already being actively pursued in VSCC (56, 57), and our study suggests that stromal TAMs may serve as a biomarker to identify a subset of VSCC that will specifically benefit from anti-VEGF treatment, as well as additional therapeutic depletion of TAMs to further support anti-angiogenic treatment.

## Supplementary Material

Supplementary Figure 1 Supplementary Figure 1 shows T cell-based immune phenotypes and spatial analysis of immune cell populations

Supplementary Figure 2 Supplementary Figure 2 shows analysis in independent validation cohorts.

Supplementary Figure 3 Supplementary Figure 3 shows expression of VEGF proteins.

Supplementary Table 1 Supplementary Table 1 summarizes immunohistochemical antibodies.

Supplementary Table 2 Supplementary Table 2 summarizes fluorescently labeled antibodies.

Supplementary Table 3 Supplementary Table 3 summarizes clinicopathological characteristics of the discovery cohort.

Supplementary Table 4 Supplementary Table 4 summarizes immunohistochemical characteristics of the discovery cohort.

Supplementary Table 5 Supplementary Table 5 shows the correlation between immune cells and HPV.
