# Supplementary material for: Immune Profiling of Vulvar Squamous Cell Cancer Discovers a Macrophage-rich Subtype Associated with Poor Prognosis
Source: Cancer Res Commun. 2024 Mar 21;4(3):861–75. doi: 10.1158/2767-9764.CRC-22-0366 (PMC10956503; doi:10.1158/2767-9764.CRC-22-0366)
Supplement: Supplementary Figure 1 — shows T cell-based immune phenotypes and spatial analysis of immune cell populations [file crc-22-0366-s01.pdf]

SUPPLEMENTARY FIGURE 1

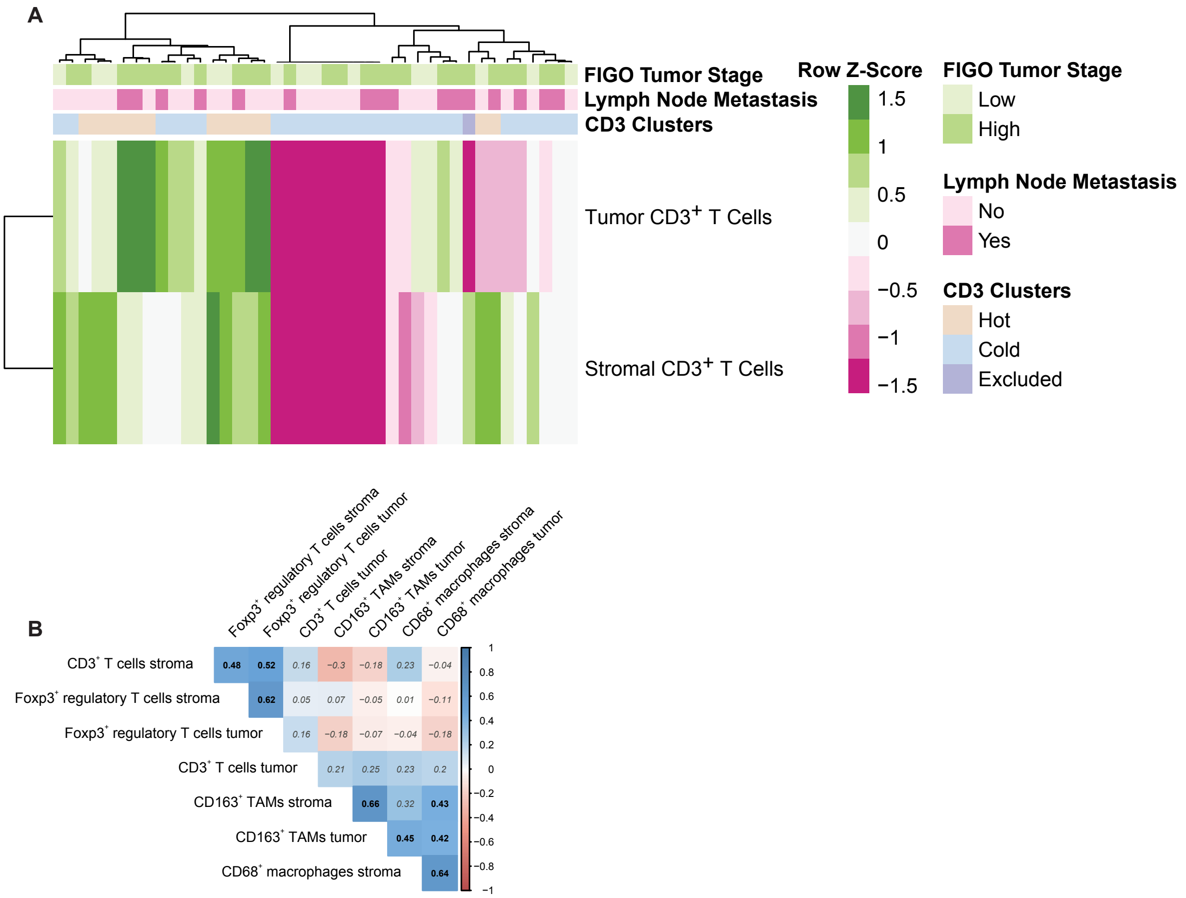

Supplementary Figure 1. T cell-based immune phenotypes and spatial analysis of immune cell populations. (**A**) Heatmap of $CD3^+$ T cells in tumor and peritumoral stroma. Unsupervised clustering of $\log_2$-transformed cell count data from 41 samples was performed based on Euclidean distance. Samples annotated as 'hot' had a pronounced T cell infiltrate in the tumor and/or peritumoral stroma; samples annotated as 'cold' had no tumor T cells; and samples annotated as 'immune excluded' had no tumor T cells but high stroma T cell counts. (**B**) CD163 immunoreactivity was assessed and counts of $CD68^+$, $Foxp3^+$, $CD3^+$ immune cells were determined. Immune cells were annotated with their spatial location and the correlation between immune cell abundance and their distribution was evaluated. The correlation matrix illustrates the relationships between immune cells and their spatial coordinates. Boxes are color-coded based on Pearson correlation coefficients, with significant correlations in bold and nonsignificant correlations in italics (Pearson p-value > 0.05).
