# Supplementary material for: Immune Profiling of Vulvar Squamous Cell Cancer Discovers a Macrophage-rich Subtype Associated with Poor Prognosis
Source: Cancer Res Commun. 2024 Mar 21;4(3):861–75. doi: 10.1158/2767-9764.CRC-22-0366 (PMC10956503; doi:10.1158/2767-9764.CRC-22-0366)
Supplement: Supplementary Figure 2 — shows analysis in independent validation cohorts. [file crc-22-0366-s02.pdf]

## SUPPLEMENTARY FIGURE 2

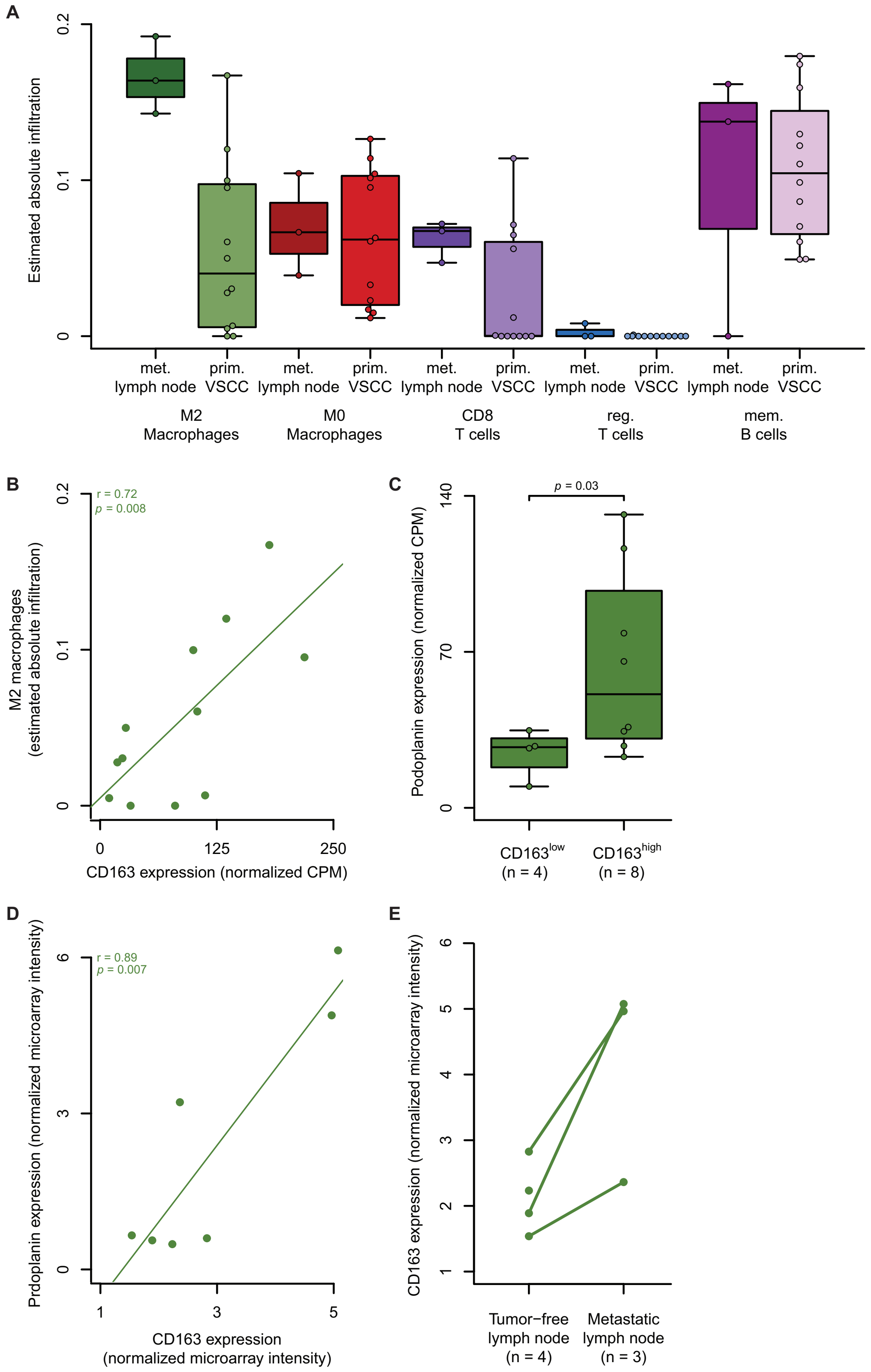

**Supplementary Figure 2. Independent cohorts validate the association between TAMs and the lymphatic system in VSCC.** (**A**) CIBERSORT-estimated immune cell type infiltration in primary (prim) VSCC and metastatic (met) lymph nodes of VSCC patients based on normalized gene expression profiles. Individual data points shown as dots overlap summary statistic boxplots with medians represented by horizontal center lines (reg, regulatory; mem, memory). (**B**) CIBERSORT-estimated M2 macrophage infiltration in primary VSCC; correlation with CD163 expression was calculated for individual samples (dots); regression line, Pearson correlation coefficient and *p*-value are shown (CPM, counts per million). (**C**) Primary VSCC cases were classified into three groups based on CD163 expression; podoplanin expression was assessed in the lower group (low) and the upper two groups (high); individual data points shown as dots overlap summary statistic boxplots with medians represented by horizontal center lines. Significance analysis by two-sided Student's t-test (CPM, counts per million). (**D**) Correlation between CD163 and podoplanin expression in lymph nodes of VSCC patients was calculated for individual samples (dots); regression line, Pearson correlation coefficient, and *p*-value are shown. (**E**) CD163 expression in matched tumor-free and metastatic lymph nodes of VSCC patients; dots represent individual samples, lines indicate match between samples (metastasis data were not available for one patient).
