# Supplementary material for: Immune Profiling of Vulvar Squamous Cell Cancer Discovers a Macrophage-rich Subtype Associated with Poor Prognosis
Source: Cancer Res Commun. 2024 Mar 21;4(3):861–75. doi: 10.1158/2767-9764.CRC-22-0366 (PMC10956503; doi:10.1158/2767-9764.CRC-22-0366)
Supplement: Supplementary Figure 3 — shows expression of VEGF proteins. [file crc-22-0366-s03.pdf]

SUPPLEMENTARY FIGURE 3

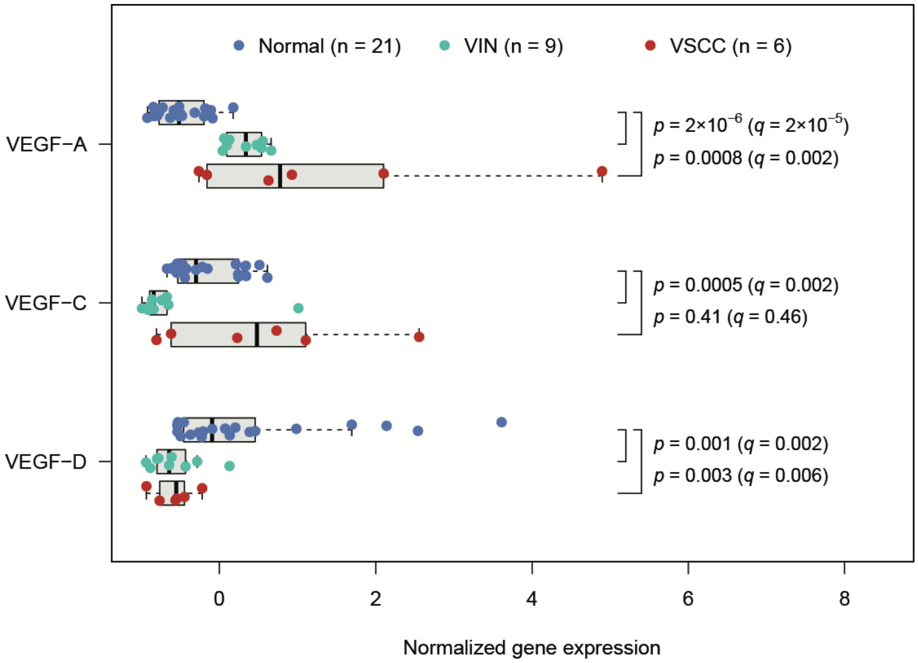

Supplementary Figure 3. Expression of VEGF proteins in VSCC development. Z-scored normalized gene expression for VEGF-A, VEGF-C and VEGF-D are derived from microarray data from normal vulvar tissue, vulvar intraepithelial neoplasia (VIN) and VSCC. Individual data points, shown as dots, overlap summary statistics boxplots with medians represented by horizontal center lines. Significance analysis by two-sided Mann-Whitney U test with Benjamini-Hochberg procedure.
