# Supplementary material for: Immune Profiling of Vulvar Squamous Cell Cancer Discovers a Macrophage-rich Subtype Associated with Poor Prognosis
Source: Cancer Res Commun. 2024 Mar 21;4(3):861–75. doi: 10.1158/2767-9764.CRC-22-0366 (PMC10956503; doi:10.1158/2767-9764.CRC-22-0366)
Supplement: Supplementary Table 1 — summarizes immunohistochemical antibodies. [file crc-22-0366-s04.pdf]

Supplementary Table 1. Antibodies for immunohistochemistry

Antigen / Name	Clone	Isotype	Dilution	Source
CD3	PS1	monoclonal mouse IgG2a	1:50	Novocastra, Newcastle Upon Tyne, UK
CD20	L26	monoclonal mouse IgG2a	1:2000	Dako, Glostrup, Denmark
CD68	PG-M1	monoclonal mouse IgG3	1:250	Dako, Glostrup, Denmark
CD163	10D6	monoclonal mouse IgG1	1:1250	Leica Biosystems, Wetzlar, Germany
Foxp3	236A/E7	monoclonal mouse IgG1	1:50	Abcam, Cambridge, UK
PD-L1	22C3	monoclonal mouse IgG1	1:25	Dako, Glostrup, Denmark
CD31	JC70A	monoclonal mouse IgG1	1:100	Dako, Glostrup, Denmark
D2-40	D2-40	monoclonal mouse IgG1	1:50	Covance, Dedham, MA, USA
Ki-67	MIB-1	monoclonal mouse IgG1	1:500	Dako, Glostrup, Denmark
p53	DQ-7	monoclonal mouse IgG2b	1:500	Dako, Glostrup, Denmark
CINtec© Histology kit for the evaluation of p16INK4a				Roche, Basel, Switzerland
