# Supplementary material for: Immune Profiling of Vulvar Squamous Cell Cancer Discovers a Macrophage-rich Subtype Associated with Poor Prognosis
Source: Cancer Res Commun. 2024 Mar 21;4(3):861–75. doi: 10.1158/2767-9764.CRC-22-0366 (PMC10956503; doi:10.1158/2767-9764.CRC-22-0366)
Supplement: Supplementary Table 2 — summarizes fluorescently labeled antibodies. [file crc-22-0366-s05.pdf]

Supplementary Table 2. Fluorescently labeled antibodies

Antigen / Name	Clone	Fluophore	Isotype	Source
CD14	M5E2	PE, APC, FITC	monoclonal mouse IgG2a	BioLegend, San Diego, CA, USA
CD80	L307.4	PE	monoclonal mouse IgG1	BD Biosciences, Heidelberg, Germany
CD86	2331 (FUN-1)	APC	monoclonal mouse IgG1	BD Biosciences, Heidelberg, Germany
CD163	GHI/61	PE	monoclonal mouse IgG1	BD Biosciences, Heidelberg, Germany
CD206	19.2	APC	monoclonal mouse IgG1	BD Biosciences, Heidelberg, Germany
EpCAM	EBA-1	PerCP-Cy5.5	monoclonal mouse IgG1	BD Biosciences, Heidelberg, Germany
HLA-DR	L243	PE/Cy7, PerCP-Cy5.5	monoclonal mouse IgG2a	BD Biosciences, Heidelberg, Germany
VEGF-A121,165,206	23410	APC	monoclonal mouse IgG2a	R&D Systems, Minneapolis, USA
VEGF-A121,165,189	VG-1	unconjugated	monoclonal mouse IgG1	Abcam, Cambridge, UK
Goat anti-mouse antibody	-	Alexa Fluor 488	IgG (H+L)	Invitrogen, Karlsruhe, Germany
Goat anti-mouse antibody	-	Alexa Fluor 750	IgG (H+L)	Invitrogen, Karlsruhe, Germany
