# Supplementary material for: Immune Profiling of Vulvar Squamous Cell Cancer Discovers a Macrophage-rich Subtype Associated with Poor Prognosis
Source: Cancer Res Commun. 2024 Mar 21;4(3):861–75. doi: 10.1158/2767-9764.CRC-22-0366 (PMC10956503; doi:10.1158/2767-9764.CRC-22-0366)
Supplement: Supplementary Table 3 — summarizes clinicopathological characteristics of the discovery cohort. [file crc-22-0366-s06.pdf]

Supplementary Table 3. Clinicopathological characteristics of the discovery cohort

**Variable**		**Value** [median ± MAD (range)]
Age (yrs)		64 ± 12 (33 - 85)
		**Value** [median (95% CI)]
Follow-up time (yrs)		6.79 (2.52 - 8.52)
		**Value** [n (%)]
Tumor stage (TNM)	pT1a	1 (2)
	pT1b	21 (43)
	pT2	20 (41)
	pT3	7 (14)
Tumor stage (FIGO)	I	19 (39)
	II	12 (25)
	III	11 (22)
	IV	7 (14)
Tumor grade	1	4 (8)
	2	34 (69)
	3	11 (23)
Depth of stromal invasion	≤ 1mm	1 (2)
	> 1mm	38 (78)
	ND	10 (20)
Lymph node involvement	Present	17 (35)
	Absent	32 (65)
Metastasis	Present	3 (6)
	Absent	46 (94)
Local surgical treatment	Wide local excision	3 (6)
	Partial vulvectomy	14 (29)
	Vulvectomy	32 (65)*
Tumor-free margins	Present	42 (86)
	Absent	7 (14)
Lymph node dissection	Sentinel	2 (4)
	Inguinal (uni-, bilateral)	47 (96)**
Radiation therapy	Vulvar	12 (25)
	Vulvar & inguinal	10 (20)***
	No radiation	27 (55)
Chemotherapy	Concurrent chemoradiation	1 (2)
	No chemotherapy	48 (98)
Disease status	No evidence of disease	32 (65)
	Recurrent disease	16 (33)
	ND	1 (2)
Outcome	Alive	27 (55)
	Death	22 (45)

CI, confidence interval; MAD, median absolute deviation; ms, months; ND, not determined; yrs, years
*, includes 5 cases of pelvic exenteration; **, includes 4 cases of pelvic/paraaortic lymphadenectomy
***, includes 3 cases of pelvic/iliac radiation therapy
