# Supplementary material for: Immune Profiling of Vulvar Squamous Cell Cancer Discovers a Macrophage-rich Subtype Associated with Poor Prognosis
Source: Cancer Res Commun. 2024 Mar 21;4(3):861–75. doi: 10.1158/2767-9764.CRC-22-0366 (PMC10956503; doi:10.1158/2767-9764.CRC-22-0366)
Supplement: Supplementary Table 4 — summarizes immunohistochemical characteristics of the discovery cohort. [file crc-22-0366-s07.pdf]

Supplementary Table 4. Immunohistochemical characteristics of the discovery cohort

**Variable**		**Value** [median ± MAD (range)]
$CD3^+$ T cells (n)	Intratumoral	11 ± 16 (0 - 143)
	Stroma	72 ± 107 (0 - 1478)
$CD20^+$ B cells (n)	Intratumoral	0 ± 0 (0 - 3)
	Stroma	2 ± 2.97 (0 - 399)
$Foxp3^+$ T cells (n)	Intratumoral	8.06 ± 5.93 (0 - 50.83)
	Stroma	57.46 ± 33.16 (0.84 - 219.44)
$CD68^+$ macrophages (n)	Intratumoral	0.5 ± 2.46 (0 - 32)
	Stroma	10.5 ± 15.57 (0 - 378)
$CD163^+$ TAMs (immunoreactivity)	Intratumoral	0.97 ± 0.76 (0 - 7.85)
	Stroma	5.16 ± 4.29 (0.06 - 19.29)
Lymph vessel density (LVD, n)	Stroma	6.68 ± 4.33 (1.33 - 53)
Blood vessel density (BVD, n)	Stroma	13.38 ± 6.99 (4.2 - 54.74)
**Variable**		**Value** [n (%)]
Ki-67 immunoreactivity	High	12 (25)
	Low	28 (57)
	ND	9 (18)
p53 immunoreactivity	Positive	14 (29)
	Negative	27 (55)
	ND	8 (16)
$p16^{INK4a}$ immunoreactivity	Positive	32 (65)
	Negative	17 (35)
High-risk HPV	Present	12 (25)
	Absent	31 (63)
	ND	6 (12)
Lymphovascular invasion (LVI)	Present	21 (43)
	Absent	28 (57)
Blood vessel invasion (BVI)	Present	5 (10)
	Absent	44 (90)

MAD, median absolute deviation; n, number; ND, not determined; TAMs, tumor-associated macrophages
