# Supplementary material for: Immune Profiling of Vulvar Squamous Cell Cancer Discovers a Macrophage-rich Subtype Associated with Poor Prognosis
Source: Cancer Res Commun. 2024 Mar 21;4(3):861–75. doi: 10.1158/2767-9764.CRC-22-0366 (PMC10956503; doi:10.1158/2767-9764.CRC-22-0366)
Supplement: Supplementary Table 5 — shows the correlation between immune cells and HPV. [file crc-22-0366-s08.pdf]

Supplementary Table 5. TAMs are not correlated with VSCC subtypes

Variable	Tumor			Stroma		
	high	low	*p*-value	high	low	*p*-value
	CD163^{+} TAMs (n=40)					
HPV-associated VSCC	6	7	0.5	8	5	1
HPV-independent VSCC	17	10		15	12	

n, number
